# Structural basis for phospholipase A_2_-like toxin inhibition by the synthetic compound Varespladib (LY315920)

**DOI:** 10.1038/s41598-019-53755-5

**Published:** 2019-11-20

**Authors:** Guilherme H. M. Salvador, Antoniel A. S. Gomes, Wendy Bryan-Quirós, Julián Fernández, Matthew R. Lewin, José María Gutiérrez, Bruno Lomonte, Marcos R. M. Fontes

**Affiliations:** 1 Departamento de Física e Biofísica, Instituto de Biociências, Universidade Estadual Paulista (UNESP), Botucatu, SP Brazil; 2 Instituto Clodomiro Picado, Facultad de Microbiología, Universidad de Costa Rica, San José, Costa Rica; 3 Center for Exploration and Travel Health, California Academy of Sciences, San Francisco, CA 94118 USA

**Keywords:** X-ray crystallography, Biophysical chemistry

## Abstract

The World Health Organization recently listed snakebite envenoming as a Neglected Tropical Disease, proposing strategies to significantly reduce the global burden of this complex pathology by 2030. In this context, effective adjuvant treatments to complement conventional antivenom therapy based on inhibitory molecules for specific venom toxins have gained renewed interest. Varespladib (LY315920) is a synthetic molecule clinically tested to block inflammatory cascades of several diseases associated with elevated levels of secreted phospholipase A_2_ (sPLA_2_). Most recently, Varespladib was tested against several whole snake venoms and isolated PLA_2_ toxins, demonstrating potent inhibitory activity. Herein, we describe the first structural and functional study of the complex between Varespladib and a PLA_2_-like snake venom toxin (MjTX-II). *In vitro* and *in vivo* experiments showed this compound’s capacity to inhibit the cytotoxic and myotoxic effects of MjTX-II from the medically important South American snake, *Bothrops moojeni*. Crystallographic and bioinformatics analyses revealed interactions of Varespladib with two specific regions of the toxin, suggesting inhibition occurs by physical blockage of its allosteric activation, preventing the alignment of its functional sites and, consequently, impairing its ability to disrupt membranes. Furthermore, based on the analysis of several crystallographic structures, a distinction between toxin activators and inhibitors is proposed.

## Introduction

Venomous snakes of medical importance are widely distributed in tropical and sub-tropical countries from Africa, Asia, Latin-America and Oceania, being responsible for an estimated 420,000 to 1,800,000 envenomings every year^1,2^. The most affected victims are usually agricultural workers from rural areas, hunters and children who do not have adequate protective clothing, secure housing or education about other protective measures that can help prevent snakebites^3^. In 2017, the World Health Organization (WHO) added snakebite envenoming to its list of neglected tropical diseases, with a focus on strategies to reduce the burden and control the effects of these envenomings^4,5^. Further, toxin-specific treatments that could be administered at the time of a bite, or where conventional serum therapies are not adequately effective, remain elusive^6^.

In Latin America, *Bothrops* species are responsible for the majority of snakebite envenomings, followed by *Crotalus* species^7–9^. Accidents involving the former are characterized by drastic local effects, often due to the action of myotoxic proteins causing muscle necrosis and, in severe cases, tissue loss, or even limb amputation and disability of the victim^10–12^.

Venoms from *Bothrops* snakes are composed of a set of proteins that have diversified functions^13–15^. Among venom components, several variants of secreted phospholipases A_2_ (PLA_2_s) are common in these venoms. Asp49-PLA_2_s display catalytic activity, and the basic variants are typically myotoxic, in contrast to their acidic counterparts which generally lack myotoxic activity. On the other hand, the Lys49-PLA_2_-like proteins lack catalytic activity, but induce myotoxicity. By acting in synergy between themselves^16^ and with proteinases^17^, myotoxic Asp49-PLA_2_s and Lys49-PLA_2_-like proteins are the main venom components responsible for local myonecrosis in *Bothrops *envenomings. Asp49-PLA_2_ toxins are calcium-dependent small proteins (~13 kDa) with secondary structure composed by three α-helices, one anti-parallel β-sheet and a calcium binding loop^18,19^. Their myotoxic activity depends on the hydrolysis of membrane phospholipids, as chemical modification of the catalytic His48 abolishes both catalysis and myotoxicity^16^. On the other hand, Lys49-PLA_2_-like myotoxins share the same general scaffold as Asp49-PLA_2_s, but display myotoxicity in the absence of catalysis. A hypothesis on the mechanism of myotoxicity induced by Lys49-PLA_2_-like toxins was recently presented, and involves several steps. It is proposed that initiation occurs by the binding of a fatty acid into the hydrophobic channel of the toxin, followed by allosteric activation and alignment of functional sites. These functional sites interact with the cell membrane via a membrane docking site (MDoS), and by the disruption of membrane integrity by the action of a membrane disrupting site (MDiS), ultimately resulting in cell necrosis^20–22^.

The cornerstone for the treatment of snakebite envenomings is antivenom administration, but scarce availability and considerable costs frequently limit the access to these life-saving antidotes in many impoverished rural areas of the world^12^. Moreover, since antivenoms have to be administered in health facilities, there is often a delay in antivenom infusion owing to the usual difficulties of patients to reach these facilities in many rural settings. In search of alternative and effective adjuvant treatments to complement the conventional antivenom therapy, *in vitro* and *in vivo* studies have tested a number of inhibitors against diverse crude venoms, or isolated toxins such as PLA_2_s^23–32^, monoclonal antibodies^33–36^ and synthetic molecules^37–48^. Ideally, these novel antidotes could be used in the field rapidly after the onset of envenoming, hence halting the deleterious action of venom toxins in the tissues. In order to understand how these inhibitors block the action of toxins, protein crystallography has been employed as a powerful tool to understand the inhibitory mechanisms of a variety of small ligands toward PLA_2_ toxins^6,21,41,44,45,47,49,50^.

Among a wide variety of molecules capable of inhibiting PLA_2_ enzymes^51,52^, one potent inhibitor of human secreted group IIA PLA_2_s is Varespladib (LY315920)^53^. This synthetic molecule was developed and clinically tested for the purpose of blocking inflammatory cascades of several diseases associated with elevated sPLA_2_ levels such as rheumatoid arthritis, sepsis and acute coronary syndrome^54^. Partly on the basis of homology between the human group IIA PLA_2_ and PLA_2_ toxins found in snake venoms, Varespladib was tested against a large panel of whole venoms from medically important snakes from different continents and potent inhibition of their PLA_2_ activity was found^42^. Inhibition has been also studied using several isolated PLA_2_ toxins, including a myotoxin isolated from the venom of *Bothrops asper*^48^.

In this communication, we describe the crystal structure of MjTX-II, a PLA_2_-like toxin isolated from *Bothrops moojeni*^55,56^ co-crystallized with Varespladib, revealing two inhibitor molecules interacting with the hydrophobic channel of the dimeric assembly of this toxin, and a comprehensive analysis of other crystal structures of bothropic PLA_2_-like toxins/inhibitor complexes using bioinformatics approaches. Furthermore, we performed *in vitro* and *in vivo* studies to assess the inhibition of toxic effects of MjTX-II by Varespladib. Taken together, the data presented hereby provide a molecular basis to understand such inhibition. This comparative analysis of crystallographic structures of PLA_2_-like toxins/inhibitors contributes to organize and classify the different inhibition models for toxic effects of PLA_2_-like toxins by different molecules into three main classes.

## Results

### Varespladib inhibits the *in vivo* myotoxicity and *in vitro* cytotoxicity of MjTX-II

**Figure 1:**
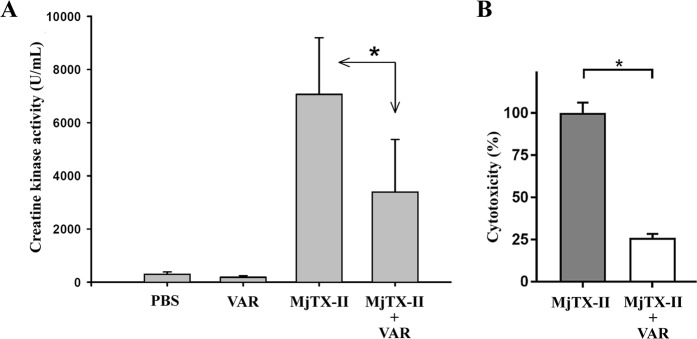
Myotoxic and cytotoxic activities of MjTX-II in mice and cultured C_2_C_12_ myoblasts, respectively, and their inhibition by preincubation with Varespladib. (**A**) Mice were injected by intramuscular route with toxin alone (50 μg, in 100 μL of PBS), or preincubated for 15 min with Varespladib (VAR) at a final concentration of 400 μM. Control groups were injected with PBS alone or Varespladib alone, respectively. After 3 hr, blood was obtained and the plasma creatine kinase (CK) activity was determined, as described in Methods. Each bar represents the mean ± SD of 4–5 mice per group. (**B**) Cells were exposed to the toxin alone (20 μg, in 150 μL of medium), or preincubated for 15 min with Varespladib (VAR) at a final concentration of 400 μM. After incubating the cells for 3 hr at 37 °C, an aliquot of supernatant was assayed for lactate dehydrogenase (LDH) activity, as described in Methods. Cytotoxicity is expressed as percentage, considering the LDH activity of cells exposed to medium with 0.1% Triton X-100, or to medium alone, as 100% and 0%, respectively. Each bar represents the mean ± SD of three replicates. Statistically significant (p < 0.05) differences between values obtained with the toxin alone or the toxin preincubated with Varespladib are indicated by an asterisk.

As typical of Lys49 PLA_2_-like toxins, the intramuscular injection of 50 µg of MjTX-II in mice caused a prominent elevation of plasma creatine kinase activity, indicative of skeletal muscle necrosis (Fig. 1A). This increment was reduced by nearly 50% when the toxin was preincubated with Varespladib, a statistically significant (*p* < 0.05) difference. Preincubation with Varespladib was also able to significantly inhibit the cytotoxic action of MjTX-II on C_2_C_12_ myoblasts *in vitro*, reducing the release of lactate dehydrogenase to the medium by nearly 75% in comparison to the effect of toxin alone (Fig. 1B).

### Crystallographic structure of MjTX-II/Varespladib

MjTX-II/Varespladib crystallographic structure has similar folding compared to group II PLA_2_s proteins previously solved, showing a dimeric configuration with seven disulfide bridges in each protomer^18,19^.

**Figure 2:**
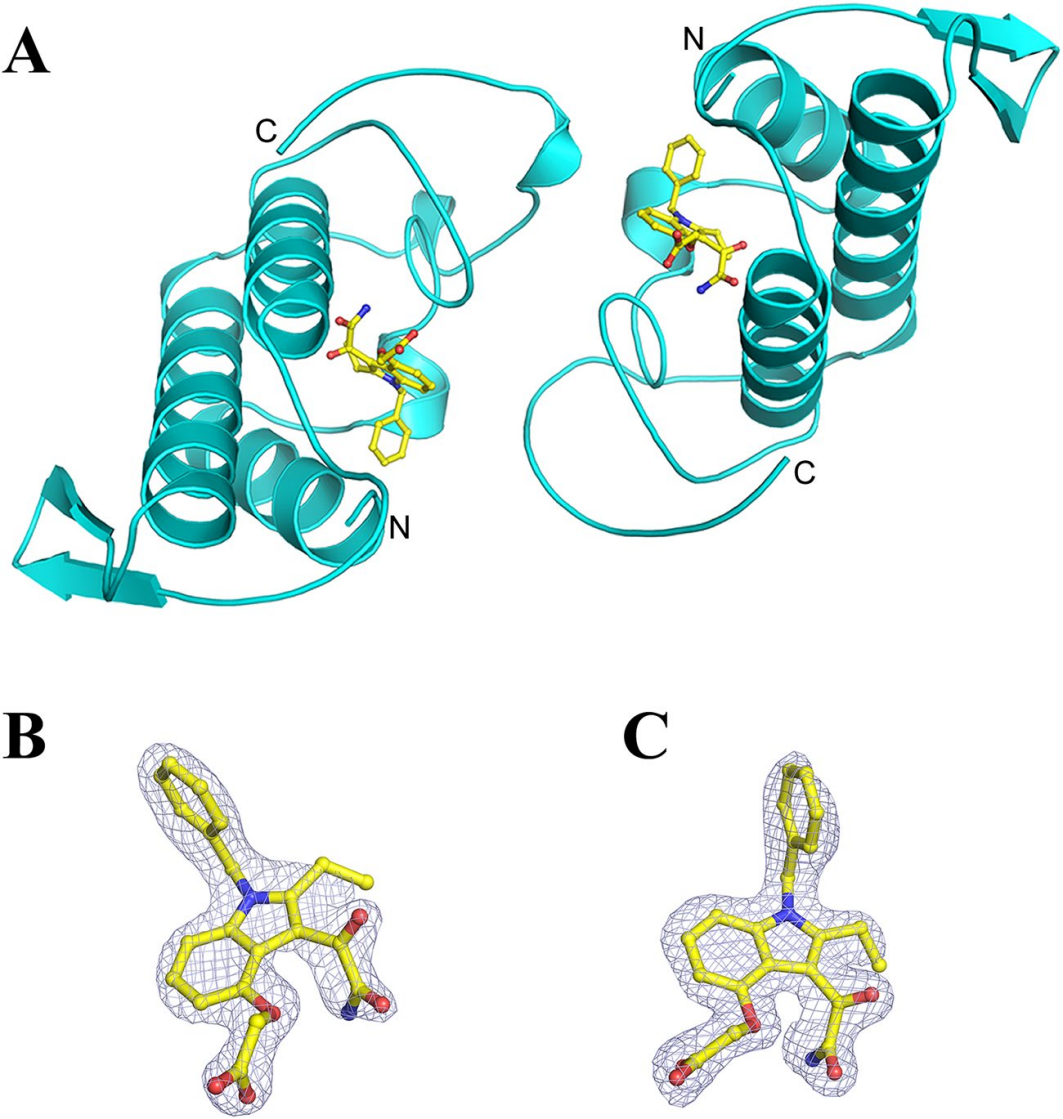
Crystal structure of the complex MjTX-II/Varespladib. (**A**) The overall structure of the complex is depicted as cartoon representation (cyan) and the inhibitor molecules are represented as sticks (yellow). (**B**) Omit electron density map (coefficients 2|F_obs_| − |F_calc_|) corresponding to Varespladib bound to monomer A and (**C**) Varespladib bound to monomer B. The maps corresponding to inhibitor molecules are contoured at 1.0 σ.

MjTX-II/Varespladib crystals belong to P2_1_ space group and diffracted up to 1.75 Å resolution. Refined data converge to an R_cryst_ of 22.42% (R_free_ = 23.73%) with a dimeric final model composed of two Varespladib molecules, two DMSO molecules and 93 solvent molecules (Fig. 2). Due to the lack of electron density, atoms from side chains of following residues are not modeled in chain A: Lys7, Lys16, Lys53, Lys69, Lys78, Lys80, Lys116, Lys128, and in chain B: Lys7, Lys36, Lys53, Lys69, Lys70, Lys78, Lys128 and Lys129.

Refinement statistics information for complex crystal structure is described in Table 1. The final model of the complex MjTX-II/Varespladib was deposited in RCSB PDB data bank, under the code 6PWH.

**Table 1:** X-ray data collection and refinement statistics for MjTX-II/Varespladib structure.

Space Group	P2_1_
Unit Cell (Å, °)	a = 54.9; b = 37.2; c = 68.7; β = 114.4
Resolution range (Å)	31.28 – 1.75 (1.81–1.75)
Unique reflections	25503 (2439)
Multiplicity	4.4 (3.8)
Completeness (%)	99.7 (98.7)
Mean *I/σ* (*I*)	16.5 (2.97)
Molecules in ASU	2
Matthews coefficient V_M_ (Å^3^Da^−1^)	2.30
*R*_*merge*_^a^ (%)	7.1 (42.2)
Reflections used in refinement	25476 (2431)
Reflections used for *R*_*free*_	1253 (116)
*R*_*work*_	21.22 (29.06)
*R*_*free*_	23.25 (27.76)
Number of non-hydrogen atoms	
Protein	1887
Water	93
Ligands	64
Varespladib/CC	2/0.92
Average B-factor	
Overall	57.04
Macromolecules	57.58
Ligands	48.75
Solvent	51.83
Ramachandran Plot (**%**)^**b**^	
Favored	94.17
Allowed	5.0
Outliers	0.83
Rotamer outliers (%)	0
C_β_ outliers	0
Clashscore	6.44
RMS (bonds) (Å)	0.004
RMS (angles) (°)	1.00

Numbers in parenthesis are for the highest resolution shell.

^a^*R*_*merge*_ = Σ_*hkl*_[Σ_i_(*I*_*hkl*,*i*_ − <*I*_*hkl*_> |)]/Σ_*hkl*_, <*I*_*hkl*_> , where *I*_*hkl*,*i*_ is the intensity of an individual measurement of the reflection with Miller indices *h*, *k* and *l*, and <*I*_*hkl*_> s the mean intensity of that reflection. Calculated for *I* > *−3σ* (*I*). ^b^Calculated with MolProbity program.

### Molecular dynamics simulations

**Figure 3:**
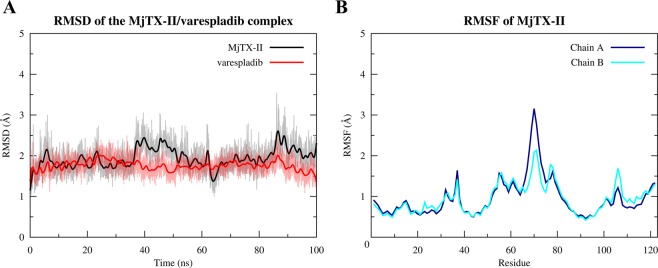
Molecular dynamic simulation of the MjTX-II/Varespladib complex. (**A**) RMSD for MjTX-II backbone atoms (black line) and Varespladib non-hydrogen atoms (red line). (**B**) Backbone atoms RMSF for MjTX-II chains A (dark blue line) and B (cyan line).

The MjTX-II/Varespladib complex presented a high stability as shown by MD simulations, evidenced by the RMSD calculations of MjTX-II backbone and Varespladib non-hydrogen atoms which present average values of 1.94 ± 0.27 Å and 1.76 ± 0.18 Å, respectively (Fig. 3A). In agreement with this, RMSF calculations of the backbone atoms of MjTX-II showed values below 5 Å (Fig. 3B).

Table 2 shows the prevalence of contacts of the toxin’s residues with Varespladib molecules below 4.5 Å. The majority of the residues presented interactions above 80% of the time simulation, except residues Gly32 and Tyr52 from both monomers that interacted between 50% and 80% of the time simulation. Furthermore, Tyr121 residue of both monomers and Leu122 of monomer B presented the lowest values, interacting with Varespladib less than 20% of the time simulation.

**Table 2:** Prevalence of contacts, expressed as percentage, of the MjTX-II residues from monomers A and B forVarespladibbelow 4.5 Å along the MD simulations.

Varespladib 1		Varespladib 2	
**Residue**	**Percentage (%)**	**Residue**	**Percentage (%)**
Leu2A	100	Leu2B	100
Leu5A	100	Leu5B	100
Gly6A	99.99	Gly6B	100
Ile9A	100	Ile9B	100
Pro18A	99.88	Pro18B	99.98
Ala19A	99.98	Ala19B	100
Tyr22A	100	Tyr22B	100
Gly23A	100	Gly23B	100
Asn28A	99.94	Asn28B	99.94
Cys29A	100	Cys29B	100
Gly30A	100	Gly30B	100
Val31A	99.23	Val31B	99.95
Gly32A	78.16	Gly32B	51.22
Cys45A	100	Cys45B	100
His48A	100	His48B	100
Lys49A	99.60	Lys49B	99.65
Tyr52A	63.17	Tyr52B	60.47
Val102A	99.16	Val102B	99.40
Leu106A	100	Ley106B	99.99
Tyr121B	14.79	Tyr121A	9.35
Pro125B	100	Pro125A	100
Phe126B	97.19	Phe126A	99.85

It is interesting to note that Varespladib molecules interacted with residues considered important for the myotoxic activity of this class of proteins, including Helix-I, MDiS, and the hydrophobic channel. The Helix-I residues Leu2 and Leu5 from both monomers were in contact with Varespladib molecules during the entire simulation time, while the Phe126 residue of both monomers interacted almost 100% of the time. Furthermore, Varespladib molecules showed a maximum percentage interaction with the His48 residues from both monomers of the MjTX-II. The binding of Varespladib to MjTX-II partially buried its MDiS region, as shown by the comparison between SASA (solvent-accessible surface area) values of MjTX-II/fatty acid (MjTX-II/myristic acid, PDB id 6B80) (1040.76 Å^2^) and MjTX-II/Varespladib (834.56 Å^2^) structures. In addition, the stability of the complex was also evidenced by the favorable ΔG of −44.98 ± 7.17 kcal/mol predicted by MM-PBSA calculation.

## Discussion

### Inhibition of PLA_2_-like toxins by varespladib

Varespladib has been reported to inhibit the enzymatic and toxic actions of a variety of snake venom PLA_2_s^42,57^, including a number of enzymes that display myotoxic activity^48^. Varespladib was able to reduce muscle damage when preincubated with the myotoxic enzymes before injection, as well as when the drug was administered soon after enzyme injection^48^. However, no information was available until now on the possible inhibition of catalytically-inactive PLA_2_-like myotoxins by this synthetic compound. In the present study, functional assays performed in mice and in cell culture demonstrated that Varespladib interacts with MjTX-II leading to a significant inhibition of its myotoxic and cytotoxic effects. In the absence of catalytic activity by MjTX-II, this finding suggests that Varespladib is able to interfere with the interaction of MjTX-II with the membrane or that it reduces its ability to disrupt plasma membrane integrity in muscle cells.

Varespladib appeared to be more effective in neutralizing the cytotoxic effect *in vitro* (75%) than the myotoxic action *in vivo* (50%), at the same inhibitor concentration (400 µM, selected from previous studies on the inhibition of catalytically-active PLA_2_s)^48^. It is likely that MjTX-II has a higher affinity for its target on mature muscle cells, compared to the myoblast cell line in culture, since an increase in susceptibility to the action of Lys49-PLA_2_-like myotoxins has been previously demonstrated to occur during the differentiation of the C_2_C_12_ myogenic cell line^58^. Therefore, differences in the affinity of MjTX-II to membrane sites in mature muscle cells *in vivo* and myoblasts *in vitro* may explain the inhibition results obtained. Our observations for inhibition of myotoxicity by Varespladib led us to focus on elucidating the molecular basis of this neutralizing interaction by using co-crystallization and MD simulation approaches, which are valuable tools to explore the mechanisms of toxicity by PLA_2_-like proteins, and to unravel the diverse modes of inhibition exerted by different small compounds. In the light of the high abundance in many viperid snake venoms of PLA_2_-like toxins devoid of enzymatic activity, but capable of inducing myonecrosis, our findings underscore that Varespladib is not only effective in the inhibition of catalytically-active toxic PLA_2_s, but also of these PLA_2_-like proteins, hence expanding the potential therapeutic usefulness of this inhibitor.

### Structural evidences for inhibition of PLA_2_-like toxins by varespladib

A mechanism to explain the myotoxic activity of Lys49 PLA_2_-like toxins was proposed recently. This model underscores the importance of interaction of fatty acid molecules in the hydrophobic channel for the initial steps of the mechanism. Essentially, after fatty acids location into the hydrophobic channel, the monomers are reoriented, resulting in toxin activation and, subsequently, the toxin docks and disrupts the integrity of the plasma membrane^20,21,59^. In this type of toxin, membrane disruption is completely independent of phospholipids enzymatic hydrolysis.

**Figure 4:**
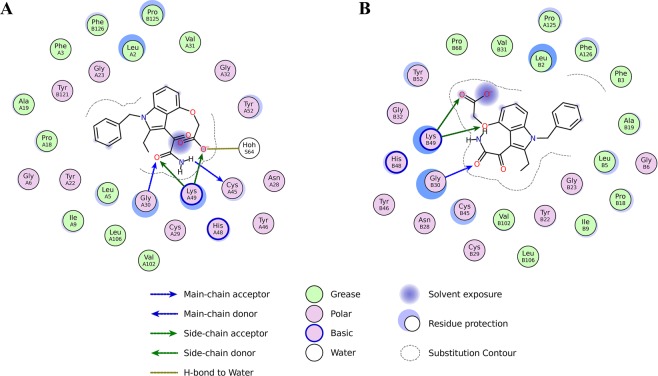
Schematic representation of the interaction of Varespladib molecules with MjTX-II. (**A**) Interaction of Varespladib with monomer A. (**B**) Contacts of Varespladib with monomer B. The figure was made using *Lidia* extension from Coot v.0.8.9^67^.

The crystal structure of the complex MjTX-II/Varespladib reveals the presence of inhibitor molecules in the hydrophobic channel of the toxin (Fig. 4), interacting particularly with His48 and Lys49 residues. The structural importance of these residues was also observed by MD simulation, in which they were found to interact with the ligand during almost the whole time (Table 2). Therefore, binding of Varespladib into the toxin’s hydrophobic channel is likely to prevent the membrane’s fatty acids binding and, consequently, precludes the structural alignment of the functional MDoS and MDiS regions, resulting in reduction of toxicity. Another structural feature observed was the particular dimeric assembly of MjTX-II/Varespladib when compared to other PLA_2_-like toxins. The superposition between dimeric proteins reveals a high distortion in their dimeric assembly, expressed by the high value of the Euler roll angle^21^ and RMSD values (Table 3). When compared to other PLA_2_-like toxins, the distorted conformation observed in the crystal structure of MjTX-II/Varespladib is also observed in the crystal structure of MjTX-II/rosmarinic acid (MjTXII/RA) and MjTX-II/acetylsalicylic acid (MjTX-II/ASA)^50^, suggesting that this structural conformation may be related to the inactive structure of MjTX-II and also shedding light on how the inhibitors can influence the structure of the toxin.

**Table 3:** Analysis of functional and binding sites of ligands for PLA_2_-like toxins from *Bothrops* snakes.

	PDB id	Toxin	RMSD (Å)^§^	Roll Angle (°)	MDoS distance (Å)	MDiS distance A (Å)	MDiS distance B (Å)	Binding sites	Inhibitor class
Toxin + Inhibitor	3HZW	BthTX-I BPB	4.13	170	13.9	9.7	6.2	Hydrophobic channel in monomer A and B	1
	2OK9	PrTX-I BPB	5.03	181	15.5	4.5	5.3	Hydrophobic channel in monomer A and B	1
	1Y4L	BaspTX-II Suramin	4.67	174	14.1	4.6	4.8	Hydrophobic channel in monomer A and B	1
	6PWH	MjTX-II Varespladib	-	163	21.1	4.8	4.5	Hydrophobic channel in monomer A and B andMDiS from both monomers	1, 2
	3QNL	PrTX-I Rosmarinic Acid	4.54	173	14.5	4.8	5.2	Hydrophobic channel entrance and MDiS from monomer B	1, 2
	4WTB	BthTX-I Zinc	4.22	142	16.8	10.3	4.7	Hydrophobic channel and MDiS from both monomers	1, 2
	6DIK	BthTX-I Chicoric Acid	4.69	180	15.4	5.0	5.1	Hydrophobic channel entrance and MDiS from monomer B	1, 2
	4YU7	PrTX-I Caffeic Acid	5.00	177	14.9	5.0	4.7	MDoS	2
	4YZ7	PrTX-I Aristolochic Acid	4.98	181	14.8	4.7	4.8	MDiS from monomer A	2
	6MQD	MjTX-II Rosmarinic Acid	0.40	166	19.3	4.7	4.8	MDiS from monomer B	2
	6CE2	MjTX-I Suramin	4.99	175	14.8	8.6	9.4	Hydrophobic channel in monomer A and B	3
	4YV5	MjTX-II Suramin	4.69	170	14.4	5.0	4.9	MDoS and MDiS simultaneously in both monomers	3
Toxin + Activator	3IQ3	BthTX-I PEG4000	5.13	178	14.5	4.7	5.2	Hydrophobic channel	—
	6B84	MjTX-II	3.73	164	13.7	4.4	4.5	Hydrophobic channel	—
	6B83	MjTX-II Fatty Acid 6	4.39	169	14.4	6.4	5.2	Hydrophobic channel	—
	6B81	MjTX-II Fatty Acid 8	3.77	167	13.7	4.9	5.0	Hydrophobic channel	—
	6B80	MjTX-II Fatty Acid 14	4.73	170	14.3	4.8	5.0	Hydrophobic channel	—
	1XXS	MjTX-II Fatty Acid 18	4.66	170	14.8	4.4	4.7	Hydrophobic channel	—
	4KF3	MjTX-II PEG4000	4.80	170	14.2	4.4	4.4	Hydrophobic channel	—
	3MLM	BnIV Fatty Acid 14	4.97	179	14.7	4.6	5.4	Hydrophobic channel	—
	4K06	MTX-II PEG4000	4.99	178	14.8	5.1	4.9	Hydrophobic channel	—
Inactive Toxin	2H8I	BthTX-I PEG400*	4.15	141	17.0	8.5	4.9	Hydrophobic channel	—
	3I3H	BthTX-I	4.15	141	17.5	9.3	4.6	—	—
	3HZD	BthTX-I	4.17	141	17.3	11.2	4.8	—	—
	2Q2J	PrTX-I	4.13	141	17.4	10.9	4.9	—	—
	4K09	BbTX-II	4.17	142	17.4	10.8	4.5	—	—
	6MQF	MjTX-II Aspirin^#^	0.44	165	21.4	4.8	4.8	Hydrophobic channel in monomer A and B	—

^§^RMSD calculated using MjTX-II/Varespladib as reference structure for superposition.

*Length of PEG400 molecule is not able to activate the toxin.

^#^Aspirin molecules bind to toxin but do not inhibit the myotoxic effects of MjTX-II.

Previously, MD simulations using the complexes MjTX-II/RA and MjTX-II/ASA showed that the distorted quaternary structural conformation of MjTX-II has some structural aspects that might be related to the myotoxic activity. The inhibitor RA remained bound to MjTX-II MDiS after 100 ns of MD simulation, preventing the access of fatty acids to the hydrophobic channel. In contrast, the ASA molecules showed an unstable interaction with MjTX-II, and left the hydrophobic channel of the toxin rapidly, allowing the further interaction of fatty acids molecules to the binding site^50^. In the present work, MD simulation of the MjTX-II/Varespladib complex showed high stability (Fig. 3A) and affinity of the inhibitor in the binding site on hydrophobic channel (Table 2), which suggests that Varespladib prevents the interaction of fatty acids with the toxin by competitive inhibition, and also preserves the distorted structure of the toxin.

The comparison of ΔG values obtained by MD simulations for MjTX-II/Varespladib and other two MjTX-II/inhibitors complexes from the three different classes (RA and suramin inhibitors - Table 3) also revealed interesting results. Although ΔG values for Varespladib and RA are in the same order of magnitude (−17.16 ± 11.67 and −44.98 ± 7.17 kcal/mol, respectively for RA e Veraspladib), the lower value for Varespladib is according to structural environment of both inhibitors. RA is partially exposed to solvent and binds superficially to MjTX-II while Varespladib binds into the hydrophobic channel of the toxin, indicating that Varespladib is an efficient inhibitor candidate for PLA_2_s-like toxins. Indeed, the very low ΔG value of MjTX-II/Suramin complex (−148.16 +/− 14.64 kcal/mol) reflects the large number of interactions and between the highly charged inhibitor and the MjTX-II.

**Figure 5:**
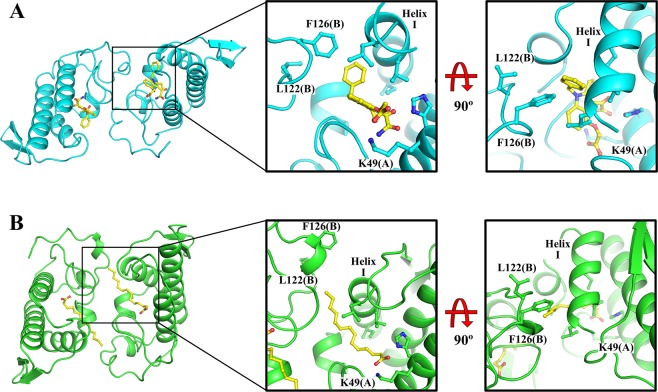
Interaction of Varespladib and myristic acid (FA14) with side chain of residues from Helix-I, hydrophobic channel and MDiS residues. (**A**) Overall crystal structure of MjTX-II/Varespladib (cyan), zoomed region of contacts of inhibitor to side chain of aminoacids from protein and the same region after 90° of rotation. (**B**) Overall of crystal structure of MjTX-II/FA14 (green), zoomed region from the interaction of fatty acid molecule with the protein and the same region after 90° of rotation.

The crystallographic structure and MD simulations analyses show that Varespladib interacts with the same site as fatty acids. Thus, the question emerges on how these molecules can bind to this site and induce contrasting effects on MjTX-II? The answer may be associated with the length of the bound molecule. Fatty acids are long chain molecules, where their tails can interact with residues from Helix-I (residues from 1 to 10) and residues from MDiS (Leu122 and Phe126 residues), hence filling the entire hydrophobic channel, and collaborating with the alignment and exposure of this functional site to the solvent (Fig. 5). On the other hand, Varespladib maintains strong interactions with the same regions (Helix-I: Leu2, Leu5, Gly6 and Ile9 residues; MDiS: Leu122 and Phe126 residues; and residues His48 and Lys49), as observed in the crystal structure (Fig. 2) and MD simulations (Table 2), occupying the hydrophobic channel internally (Fig. 5). The interaction of Varespladib with residues of the hydrophobic channel forces MDiS to remain in contact with the toxin, favoring the distorted conformation. Indeed, SASA of the MDiS region of the MjTX-II/Varespladib is about 20% lower compared to the MjTX-II/myristic acid structure (PDB id 6B80). Therefore, Varespladib may inhibit the myotoxin by two different mechanisms: (***i***) hydrophobic channel blockage and (***ii***) preventing the MDiS exposure to the solvent.

### PLA_2_-like toxins inhibitors

The activation of PLA_2_-like toxins involves important structural changes in their oligomeric structures with particular features after the binding of fatty acids into the solvent-exposed hydrophobic channel: (***i***) alignment of the membrane docking site (MDoS), where the distance between positive clusters that comprise the MDoS (Lys16, Lys20, Lys115 and Arg118) from both monomers must be ≤16 Å; (***ii***) Euler Roll angle >160° and; (***iii***) changing the distance of the MDiS hydrophobic residues Leu121 (122 for MjTX-II) and Phe125 ≤ 5 Å^21,60^. These structural features were observed in recent studies by analyses of several crystallographic structures of PLA_2_-like toxins in native form and bound with inhibitory molecules^6,25,37,41,44–46,49,50,61^.

Crystallographic structures of PLA_2_-like toxins isolated from *Bothrops* snake venoms in complex with different inhibitory ligands showed that these inhibitors may interact with the toxin in distinct regions (Table 3). Basically, three regions were identified, which coincidentally are involved in the myotoxic effects according to the structural studies: hydrophobic channel, MDoS, and MDiS regions^20,21,37,45^. To date, eight different inhibitors have been studied in complex with different bothropic PLA_2_-like toxins. Their binding region(s) are next described: (***a***) bound into the hydrophobic channel (bound to His48): *p-*bromophenacyl bromide (BPB)^41,61^, Zinc ions^37^, Suramin^44^ and Varespladib (present work); (***b***) bound in the entrance of hydrophobic channel: Chicoric acid^25^ and Rosmarinic acid^49^; (***c***) bound to the membrane disrupting site (MDiS): Zinc ions^37^, Aristolochic acid^6^, Chicoric acid^25^ and Rosmarinic acid^50^; (***d***) bound with MDoS residues: Caffeic acid^6^; (***e***) bound simultaneously to MDoS and MDiS regions and also induces protein oligomerization: Suramin^45,46^.

**Figure 6:**
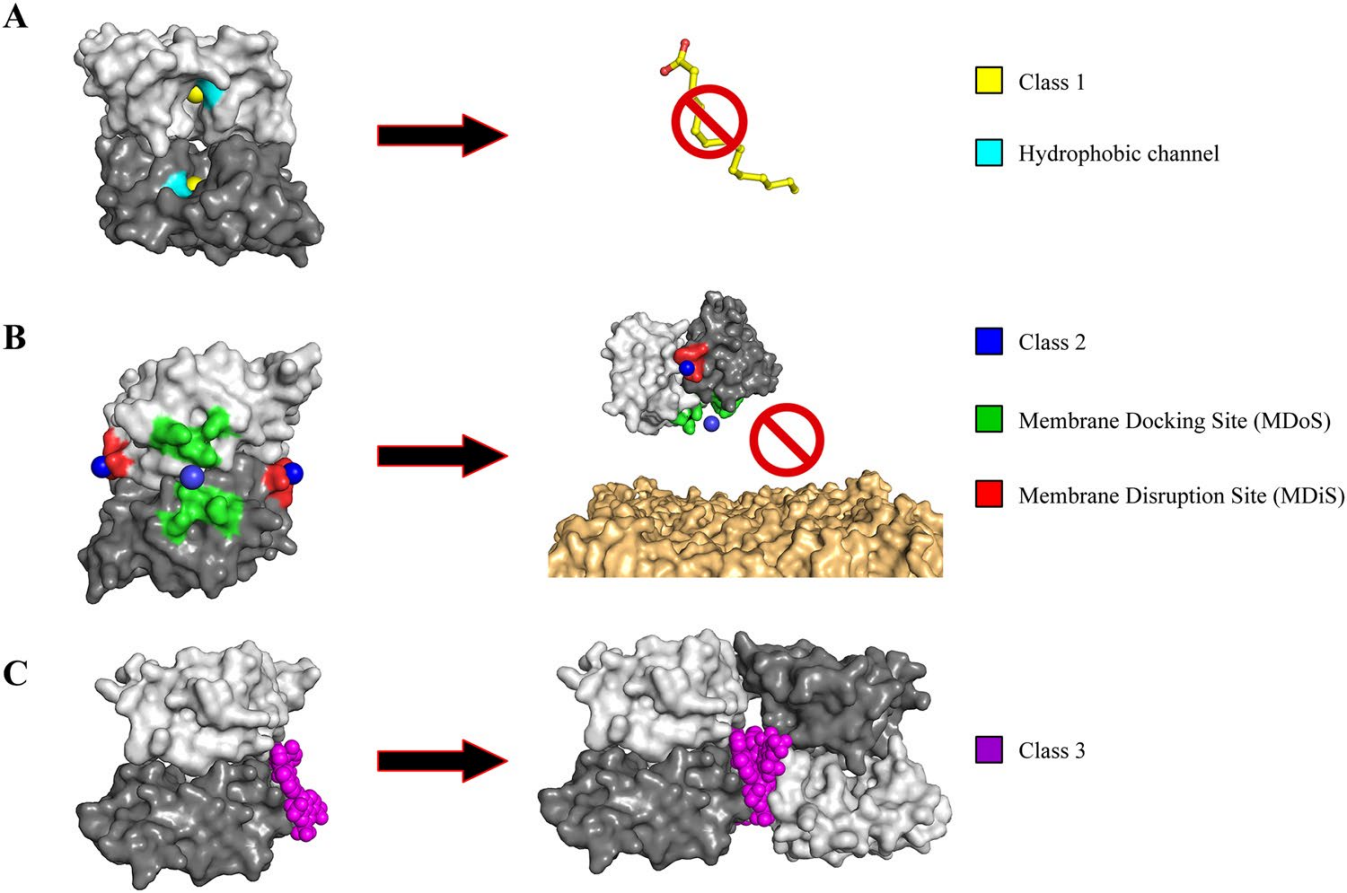
Different forms of inhibition of Lys49 PLA_2_-like toxins by inhibitors described in literature. The monomer A is showed as light gray and the monomer B is represented as dark gray (**A**) Class 1 inhibitors (yellow), which prevent the binding of fatty acids (yellow sticks - activator) to hydrophobic channel (cyan) of toxin. (**B**) Class 2 inhibitors (blue) which bind to functional MDoS (green) and MDiS (red) and prevent the interaction of toxin to membrane (orange). (**C**) Class 3 inhibitors (magenta) which induce protein oligomerization.

In summary, different inhibitors may bind to different regions of PLA_2_-like toxins of *Bothrops* sp snake venoms but may be classified into three different classes related to different mechanisms of inhibition: (***1***) Inhibitors that block the access to the hydrophobic channel for fatty acid molecules (inhibitors ***a*** and ***b***, previous paragraph), impairing the natural movement between the monomers of the dimeric structure; (***2***) inhibitors that bind to functional sites of the proteins and block MDoS and MDiS interaction with cell membrane (inhibitors ***c*** and ***d***), and (***3***) inhibitors that can induce protein oligomerization, leading to the combination of the previous mechanisms (inhibitor ***e***) (Fig. 6; Table 3).

## Conclusion

We have described functional and structural studies involving a toxin isolated from *Bothrops moojeni* venom, MjTX-II, and the inhibitor Varespladib. The results obtained by *in vitro* and *in vivo* experiments reveal that this synthetic compound is able to inhibit the myotoxic and cytotoxic effects of MjTX-II, a well characterized member of the Lys49-PLA_2_-like myotoxins, widespread among many crotaline snake species. Given that the safety of Varespladib has been already confirmed in clinical trials, present results strengthen the importance of this compound as a repurposed drug candidate to aid in the treatment of snakebite envenomings. The crystal structure and MD simulations described the interaction of Varespladib with a site similar to that of fatty acids binding and with the MDiS site, suggesting that the inhibition of MjTX-II by Varespladib is due to physical blockage of the interaction with allosteric activator molecules in the hydrophobic channel, and by restricting the movement of the MDiS of these toxins, preventing the alignment of its functional sites and, consequently, impairing its ability to disrupt the integrity of membranes. Furthermore, based on the crystallographic structure analyses, the distinction between activators and inhibitors is discussed, and the three classes of inhibition mechanisms for PLA_2_-like proteins are proposed.

## Methods

### Toxin and varespladib

The isolation of MjTX-II from crude venom of *B*. *moojeni *was previously described^50,56^. Varespladib (compound LY315920) in free acid form was kindly provided by Ophirex. Inc. (original source: ChemieTek, Indianapolis, Indiana, USA; 99.99% pure by NMR and HPLC).

### *In vivo* myotoxicity

Myotoxic activity assays were carried out in groups of five CD-1 mice (18–20 g body weight), which had access to food and water *ad libitum*, as described previously^62^. One group of mice was injected in the gastrocnemius muscle with 50 µg of toxin dissolved in 100 µL of phosphate-buffered saline (PBS, pH 7.2; 0.12 M NaCl, 0.04 M sodium phosphate). A mixture of 50 µg of toxin and 400 mM Varespladib (in 4% dimethyl sulfoxide; DMSO) was preincubated for 15 min at room temperature, and subsequently injected in another group of mice under otherwise identical conditions. Control groups of four mice were injected with either 100 µL of PBS, or 100 µL of 4% DMSO in PBS, respectively. After 3 h, blood was collected from the tip of the tail into a heparinized capillary and centrifuged. Then, plasma creatine kinase (CK) activity, expressed in U/L, was determined using a UV kinetic assay (CK-NAC UV, Wiener Lab). Animal experiments were approved by ‘Comité Institucional para el Cuidado y Uso de los Animales (CICUA, permit #084-17), Universidad de Costa Rica. After the experiments, mice were sacrificed by CO_2_ inhalation. For comparison of mean values from more than two groups, ANOVA was used followed by Tukey-Kramer tests, and differences were considered statistically significant when *p* < 0.05.

### *In vitro* cytotoxicity on C_2_C_12_ myoblasts

Murine C_2_C_12_ skeletal muscle myoblast cells (CRL-1772; American Type Culture Collection) were used in cytotoxicity assays, as previously described^16,48^. The assays were performed using a stock solution of Varespladib dissolved at 10 mM in dimethyl sulfoxide (DMSO). This solution was diluted prior to each assay with the corresponding buffers to a final concentration of 400 µM, decreasing the DMSO concentration to 4%. Control tests performed preliminarily showed that this concentration of DMSO did not result in a cytotoxic effect in the assay system. The C_2_C_12_ cell cultures were maintained as undifferentiated myoblasts at subconfluent levels in Dulbecco’s modified Eagle’s medium (DMEM, Sigma) supplemented with 10% fetal bovine serum (FBS), L-glutamine and penicillin/streptomycin. Cells (approximately 10^5^/200 µL) were added to each well of a 96-well plate and incubated at 37 °C, with a 7% CO_2_ humidified atmosphere. When cultures reached 80–90% confluence, myoblasts were incubated with either toxin alone or toxin that had been pre-incubated with Varespladib (400 µM) for 15 min at room temperature. After 3 h of cell exposure at 37 °C, an aliquot of 60 µL of each supernatant was obtained to quantify the activity of lactate dehydrogenase (LDH) release from damaged cells, using a commercial assay (LDH-P UV*AA* - Wiener Lab). The LDH activity in supernatants of cells exposed to DMEM alone, or DMEM containing 0.1% Triton X-100, was considered as 0% and 100% reference points, respectively. The assays were performed in triplicate cell cultures and the results are presented as mean ± SD. The statistical significance of differences between means of two groups was determined by the Student’s *t*-test, where values of *p* < 0.05 were considered significant.

### Crystallization and X-ray data collection

Crystallization trials for MjTX-II/Varespladib complex were performed using the purified toxin concentrated up to 10 mg.mL^−1^ (diluted in 20 mM ammonium bicarbonate pH 8.0) and Varespladib (diluted in 100% DMSO) added to obtain the molar ratio of 1:10 respectively. Crystals of the MjTX-II/Varespladib complex were obtained by conventional hanging drop vapor-diffusion method^63^ at 291 K from a drop mixture of 0.7 µL of protein, 0.3 µL Varespladib and 1 µL reservoir solution equilibrated against 500 µL reservoir. The reservoir cocktail was composed by 30% v/w polyethylene glycol (PEG) 4000, 0.1 M TrisHCl pH 8.5 and 0.2 M lithium sulfate.

A dataset was obtained using a synchrotron radiation source (MX2 station, Laboratório Nacional de Luz Sincrotron (LNLS), Campinas, Brazil) and a PILATUS 2 M detector (Dectris) using a wavelength of 1.459 Å (at 100 K). The X-ray diffraction data were collected using crystal-to-detector distance of 100 mm, oscillation of 1° per frame resulting in 250 frames. The processing was executed using the HKL2000 v.1.8.4 program package^64^ as described Table 1.

### Structure determination and refinement

The crystal structure of MjTX-II/Varespladib was solved by the molecular replacement method using the program PHASER^65^ from PHENIX package v.1.12^66^. The coordinates of the monomer A from MjTX-II (PDB access code 4KF3) was used as search model. The manual modeling and refinement process of the protein, insertion of Varespladib, DMSO and solvent molecules were performed using program Coot v.0.8.9^67^. Structural automated refinement and the general quality check of models were performed using PHENIX package v.1.12^66^ and MolProbity program (http://molprobity.biochem.duke.edu/)^68^.

### Structural comparative analysis

For the structural comparisons, the structures of the MjTX-II/Varespladib presented here and structures presented in Table 3 were used. Molecular comparisons of structures were performed using Coot v.0.8.9^67^ and PyMOL v.1.8.6^69^ programs. All structural figures were generated using PyMOL v.1.8.6^69^ program.

### Molecular dynamics (MD) simulations

MD simulations of MjTX-II in complex with Varespladib were carried out using GROMACS (Groningen Machine for Chemical Simulation) v.5.0.5^70^ under the CHARMM36 force field^71^. All the initial input parameters and Varespladib topology were generated by CHARMM-GUI webserver^72^ and the protonation of the residues was set to pH 7.0 determined by PROPKA3 server^73^. The complex was placed in a cubic box with 5 Å from the farthest atom, solvated with TIP3P water molecules and equilibrated with 100 mM of NaCl. Further, the system was minimized until reaching an energy below 100 kJ/mol/nm using the Steepest Descent algorithm. A 1-ns NVT ensemble was performed generating the velocities randomly according to Maxwell-Boltzmann distribution at temperature of 300 K using the V-rescale thermostat^74^ followed of a 1-ns NPT ensemble with Berendsen barostat^75^ at 1 bar. Both steps were performed restraining the backbone and hydrogen atoms of the protein and Varespladib, respectively. A following step of an unrestrained 100 ns NPT step was performed using the Nose-Hoover thermostat^76,77^ and Parrinello-Rahman barostat^78^. Short-range cutoffs for electrostatic and Van der Waals interactions were set to 12 Å with a force-switch function from 10 to 12 Å and hydrogen bonds were constrained using LINCS algorithm^79^.

The binding energy (ΔG) of the MjTX-II/Varespladib complex was predicted using the MM-PBSA method implemented in g_mmpbsa software^80^, collecting the frames every 500 ps for the last 10 ns of MD simulation. As MjTX-II has many charged residues, the solute dielectric constant (ε_solute_) was set to 8. In order to compare the ΔG value of Varespladib with other molecules bound to the same toxin, inhibitors from classes 1, 2 and 3 (Table 3) were selected: (***i***) Rosmarinic Acid (PDB ID: 6MQD)^50^, and (***ii***) Suramin (PDB ID: 4YV5)^45^, then ΔG values were calculated using the same protocol as for Varespladib.

The prevalence of contacts of the MjTX-II residues and Varespladib molecules were calculated selecting the residues that interacted with the ligand below a cutoff of 4.5 Å from the crystallographic structure. Further, these residues were analyzed according to the minimal distance to Varespladib molecules for each frame from MD simulations considering an interaction only when below the cutoff, then these interactions were converted to percentage and classified as weak (<50%), moderate (<80% and >50%) or strong (>80%). Root-Mean-Square Deviation (RMSD), Root-Mean-Square-Fluctuations (RMSF) and Solvent-Accessibly Surface Area (SASA) calculations were performed using built-in tools provided by GROMACS.

### Ethical statement

Experiments in mice were approved by the Institutional Committee for the Care and Use of Laboratory Animals (CICUA), Universidad de Costa Rica (permit #084-17). Animal procedures were in accordance with the guidelines for animal care prepared by the Committee on Care and Use of Laboratory Animal Resources, National Research Council, USA.
